# 

**Published:** 1911-12-30

**Authors:** 


### Buildings Contemplated and in Progress.

Wolverhampton.—Mr. A. W. Worrall, of Wolverhampton, is architect for the King Edward memorial wing at the Staffordshire General Hospital. Seven tenders have been submitted, the lowest £6,185, the highest £6,900.

Bootle.—The King Edward Memorial Committee is making a further appeal for funds to carry out much-needed additions and alterations at the Bootle Borough Hospital. The scheme, which is to cost £17,000, includes further purchase of land, erection of additional wards for fifty patients with the necessary adjuncts, and an operating theatre, a new out-patients' department, and a remodelling to provide nurses' quarters. A sum of £10,753 is already subscribed.

Birstall.—Mr. J. W. Burrows, of Birstall, is architect for a proposed extension of the Infectious and Smallpox Hospital, on behalf of the Oakwell Joint Hospital Board. Tenders are invited, and a deposit of 21s. is requested.

Prague.—It is proposed to expend a sum of £250,000 on the erection of a hospital in Lieben. Accommodation for some 550 beds will be provided.

Pontefract.—Plans were approved at an inquiry by the Local Government Board into the application by the Pontefract Joint Hospital Board for sanction to borrow £2,760. Messrs. Garside and Pennington are the architects.

### A New Handbook for Medical Students.

A Manual of Physics for Medical Students. By Hugh C. H. Candy, B.A., B.Sc., F.I.C. New Edition. Illustrated. (London: Cassell and Co., Ltd. 1911. Price 6s. net.)

For the purpose of the first professional examination of the Conjoint Board the two text-books on Physics and Chemistry, published by Messrs. Cassell, will be found not only sufficient but most suitably arranged. The manual of Physics which has just been published replaces and expands a similar volume by Mr. F. J. M. Page; considerable additions have been made to keep pace with the alterations in the syllabus of the Examining Board. A most useful section is the last one, which deals with the practical side of the subject and instructs the student in the actual performance of physical measurements and experiments. This compact and clearly illustrated manual will certainly become popular among medical students. Mr. Candy has succeeded in compressing a large amount of science into a small space, but the explanations given are adequate and lucid.

### BOOKS RECEIVED.

Chatto and Windus.

"The Enzyme Treatment of Cancer." By John Beard.

T. Fisher Unwin.
"The Man-made World." Charlotte P. Gilman.

Methuen and Co., Ltd.
"Outlines of Biology." By P. Chalmers Mitchell.

Thacker, Spink and Co.
"Tropical Hygiene." By Lukis and Blackham.

Mills and Boon.
"Child Nurture." By Honnor Morten.

### Expulsion of Placenta before Fœtus.

Many years ago Sir J. J. Simpson published a collection of 141 cases of this obstetric accident, which he believed to be not so rare as was then commonly taught and supposed. He also insisted that it is not so serious and dangerous a complication as it was thought. In most of his cases unavoidable hæmorrhage was taking place, but in nineteen cases out of twenty the bleeding either ceased altogether when the placenta was expelled or at least at once diminished so much as to remove all cause for alarm. He also found that the presence or absence of flooding after the complete separation of the placenta does not seem in any degree to be regulated by the duration of time between the detachment of the placenta and the birth of the child. A further contribution to the study of this condition appeared in the *British Journal of Obstetrics and Gynæcology*, from the pen of Dr. W. D. Macfarlane, who publishes three such cases from his own records. There are several points of interest in the three cases. In all three it is probable that the placenta was partly prævia; the holes in the membranes pointed to such an occurrence, and in one patient the two preceding pregnancies had been thus complicated. In two cases the placenta was of unusual length and shape; and in all three there were numerous areas of infarction on the maternal surface. In two of the patients there was hydramnios as well; and all three had a history strongly suggestive of endometrial mischief previous to conception. As supporting the teaching of Simpson, it may be added that in no case of Dr. Macfarlane's series was there any hæmorrhage either before or after the expulsion of the placenta; and the puerperium in each case was normal.

### Gifts.

Miss Emily Ann Mills has bequeathed £1,000 to the Cancer Hospital, Fulham, and £1,000 to the Hospital for Incurables at Putney Heath.

Mrs. Mary S. P. Hyde has left a sum not exceeding £500 for the endowment of a bed in Hawick Cottage Hospital to be known as the "John and Mary Robertson" bed.

St. Bartholomew's Hospital has received £1,000 from the Drapers' Company towards the building fund.

Colonel Hope Edwards has given 50 guineas to the Infants' Hospital, Vincent Square, Westminster.

Miss Doris Voight, of Stamford Hill, has bequeathed £25 to St. Thomas's Hospital.

Mr. William H. Maude, of Leeds, has left £200 to the Leeds General Infirmary.

Mr. William Biggam, of Sunderland, has left £50 to the Sunderland and North Durham Eye Infirmary.

Mr. Chas. Bowles Hare, of Bristol, has bequeathed £1,000 to the Bristol General Hospital, £300 to the Bristol Royal Infirmary, and £100 to the Bristol Eye Hospital.

Major George Banaster has bequeathed several thousand pounds to the Ramsey Hospital, Isle of Man.

Miss Katherine Hosack Willox has left £500 to the Liverpool Sanatorium at Delamere Forest, and £500 to the Bluecoat Hospital, Liverpool.

Queen Alexandra has sent a present of pheasants to the Chelsea Hospital for Women.

Under the will of the late Mr. David Jardine, of Liverpool, the following bequests are made to institutions in that city: The Royal Infirmary, £2,000; the Seamen's Orphanage, £2,000; the Southern Hospital, £2,000; the Northern Hospital, £1,500; the Stanley Hospital, £1,500; and £1,000 each to the Bluecoat Hospital and the Children's Hospital.

Mrs. Laura Theresa Pearce, of Tunbridge Wells, has left £500 to King's College Hospital and £200 to the Tunbridge Wells General Hospital.

The Royal Dental Hospital, Leicester Square, has received £200 from the Goldsmiths' Company, and ten guineas from the Broderers' Company.

Mrs. Mary Sharman, of Sheffield, has left £250 to the Children's Hospital, Sheffield,

Mr. Charles Wardlow, of Sheffield, has made the following bequests: £500 to the Sheffield Royal Hospital, £300 to the Royal Infirmary, and £300 to the Jessop Hospital for Women.

Mr. Frederick Tytherleigh, of Teignmouth, has bequeathed £200 each to the Royal Devon and Exeter Hospital, and the West of England Eye Infirmary, Exeter; and £100 to the Axminster Cottage Hospital.

Lord Strathcona has sent a donation of £2,000 towards the building of the Queen Alexandra Wing of the British Home and Hospital for Incurables at Streatham.

Mr. Vincent F. Tufnell, of Leamington, has left £500 to the Midland Counties Home for Incurables at Leamington.

The Samaritan Free Hospital has received gifts of game from the Viscount Portman, Col. Curne, and W. B. Gladstone, Esq.

The following legacies have been received by Addenbrooke's Hospital, Cambridge: From the late Mrs. C. Saygall, £10; Mrs. Jane Wright, late of Potton, Beds, for many years a subscriber and Life Governor, one-twelfth of her residuary estate, estimated at at least £1,000; Miss Eleanor Mary Gent, late of 3 Clifton Place, Sussex Square, London, and The Moynes, Steeple Bampsted, Cambs, for many years subscriber and Life Governor, £1,000; Mrs. Ame Gray, late of 12 Guest Road, Cambridge, £20; Miss Mary Gifford, late of Willingham, Cambs, £100 (less duty).

The Rev. Henry Homer, of Rugby, has bequeathed £1,000 to the Hospital of St. Cross, Rugby, £250 to the Leicester General Infirmary, £200 to the Children's Hospital, Leicester, and £200 to the Midland Counties Home for Incurables, Leamington, besides twenty-seven other gifts of £100 each to various associations and institutions.

Mr. George Bennett, of Chesterfield, has left £50 to the Chesterfield and North Derbyshire Dispensary.

Mr. Pandelly L. Argenti has left £500 to the Middlesex Hospital, £500 to the East London Hospital for Children, Shadwell, £250 to the French Hospital in London, and £250 to St. Mary's Hospital.

Miss Mary Ann Fisher has left £100 to the York County Hospital.

### Florence Nightingale Memorial.

We have received the following contributions since those acknowledged last week. They are for a statue, unless otherwise indicated: John Marshall, Esq., £5 5s.; per John Marshall, Esq.: E. Willoughby Firth, Esq., £5 5s.; Frank Atkin, Esq., £2 2s.; Dr. Sinclair White, £1; Miss Charlotte Williams, 8s. 6d.

Further contributions may be sent to the Hon. Sec., Florence Nightingale Memorial, St. Thomas' Hospital, Albert Embankment, London, S.E., or to the Editor of the Hospital, 28 Southampton Street, Strand, London, W.C.

### Appointments.

Mr. W. E. Haigh, F.R.C.S. Eng., L.R.C.P., D.T.M., has been appointed senior resident medical officer at the General Hospital, Northampton.

Mr. W. I. Cumberlidge has been appointed assistant surgeon to the Leicester Infirmary, in the place of Mr. J. S. Sloane, appointed surgeon. Mr. Cumberlidge graduated in Arts with first-class honours at Cambridge in 1901, and qualified M.R.C.S., L.R.C.P. in 1904. Subsequently he graduated in medicine and surgery at Cambridge, and was elected a F.R.C.S. in 1907. He has held the following resident posts: House surgeon and obstetric house surgeon, St. Bartholomew's Hospital; house appointments at the London Temperance, St. Luke's, and the Royal London Ophthalmic Hospitals; and house surgeon to the Leicester Infirmary.

### The Week's Novelties.

### NOVOFORM (HEYDEN).

(E. W. Blasius, 127 Fenchurch Street, London.)

This is pulverised tetra-brom-pyro-catechin-bismuth, manufactured at the Heyden factory, near Dresden. It is a fine yellow powder, not unlike curry powder in appearance, insoluble in water, and perfectly odourless. Its germicidal and disinfecting powers are strong, and it is stated to be non-poisonous. As a dusting powder it is specially valuable on account of the fact that it does not "crust" or "scab" when applied to raw surfaces. It may be mentioned that it can be used as a deodorant for fumigating purposes by sprinkling a few grains of the powder on some coals or on a hot surface.

### FICOLAX.

(The Ficolax Manufacturing Co., 22 Graham Street, City Road, London, N.)

This is a laxative compound prepared "from figs and other vegetable ingredients." It is a very pleasant-tasting, dark ruby-red coloured fluid of a syrupy consistency, and contains, so far as we have been able to ascertain, no mineral purgatives or aperients, but is simply a flavoured decoction of figs, agreeably sweetened and blended. As such it is particularly suitable for children when a mild and gentle laxative is required. The dose of the syrup is one to two teaspoonfuls for adults, and children's doses in proportion. The price charged is low in comparison with the excellence of the syrup.

